# Teachers’ perspectives on influencing factors of children’s museum education in China: a grounded theory study

**DOI:** 10.3389/fpsyg.2026.1738887

**Published:** 2026-01-16

**Authors:** Di Wu

**Affiliations:** School of Music and Dance, Zhengzhou University of Science and Technology, Zhengzhou, China

**Keywords:** Bronfenbrenner’s ecological systems theory, Chinese museums, grounded theory, museum education for children, teachers’ perspectives

## Abstract

**Introduction:**

Museum education has become an important approach for children to acquire knowledge and engage in enjoyable learning activities. Although previous studies have examined the development of museum services, limited attention has been paid to museum education for young visitors, particularly from teachers’ perspectives.

**Methods:**

This study focused on early childhood and primary school teachers in China who had organized or implemented museum education activities. Using a grounded theory approach, semi-structured interviews were conducted to explore children’s museum education and its influencing factors. The data were analyzed from two dimensions: internal factors (including teachers’ experiences, cognitions, and emotions) and external factors (such as environmental conditions and institutional support).

**Results:**

The findings revealed that both internal and external factors jointly shape teachers’ practices and perceptions of children’s museum education. Teachers’ professional experiences and emotional engagement, along with supportive environments and institutional mechanisms, were identified as key influences on the implementation of museum education activities.

**Discussion:**

These results provide important implications for improving teacher intervention programs, developing supportive policy frameworks, optimizing the dissemination of museum education, and enhancing museum resources and services. Such efforts may further promote the sustainable development of children’s museum education.

## Introduction

Recently, an increasing amount of research has paid attention to museum education and its educational values (Gong et al., 2020). As a popular out-of-school informal learning environment, museums provide students with authentic and reliable resources and opportunities to observe and explore those resources through activities such as hands-on experiences and role play (Xu et al., 2024). With well-designed exhibitions and appropriate pedagogy, students can be empowered and their different learning needs can be satisfied (Hooper-Greenhill, 1999). It has been shown that museums are supportive of children’s learning and development by offering various types of cognitive, affective, social, and physical experiences (Tan et al., 2021; Williams et al., 2018; Garner et al., 2016; Calabrese Barton et al., 2013). Therefore, it is essential to investigate how children actually access museums and experience museum education.

There are two main avenues through which young people access museums: family visits and school trips (Wu, 2024). Although increasing attention has been paid to children’s museum visits given their various educational benefits (Young et al., 2022; Feliu-Torruella et al., 2021), researchers have placed greater emphasis on family visits, particularly on parent–child interactions in museums (Franse et al., 2020) and children’s exploratory behaviors (Weisberg et al., 2023). Callanan et al. (2017) demonstrated that children can learn from collaborative interactions with parents during museum visits. Willard et al. (2019) found that parental prompts which encouraged exploration and explanation led to distinct yet fruitful learning activities for young children. Parents can provide their children with unique learning opportunities through discussing different aspects of artifacts to varying degrees (Andre et al., 2017). When employing critical thinking questions, parents identify more artifacts and children can be highly engaged and generate more information in response (Attisano et al., 2022). However, much less is known about school-based museum visits and the role that teachers play in children’s museum learning, despite their critical position in shaping children’s museum learning experiences.

Although teachers are encouraged to implement museum education for children and young people due to its significant educational values (Shaby and Vedder-Weiss, 2020), recent literature indicates that teachers show limited intention to do so, including organizing class visits to museums, largely because of policy constraints, inadequate training, insufficient support, and limited resources (Wu, 2024; Zakaria, 2024; Chang et al., 2021; Wong and Piscitelli, 2019). Few studies have examined how children explore museums through field trips led by schools (Weisberg et al., 2023). Previous research highlighted that it was challenging to assess children’s learning, particularly among young children (Andre et al., 2017). Consequently, the understanding of children’s exploration in museums organized by schools is still limited. This study mainly focuses on school-led museum visits from teachers’ perspectives and seeks to investigate factors that influence teachers’ implementation of museum education. Therefore, the results of this study can help teachers and related educational practitioners to create quality museum education for children. Moreover, the findings can strengthen the collaboration among families, schools, and museums and provide insights for understanding children’s learning through museum education.

## Museum and young visitors

Museums are spaces that embody and integrate the values and cultures of society. The International Council of Museums (2022) defines museums as stable institutions responsible for collection, conservation, exhibition, and education. Museums provide unique opportunities for visitors to learn knowledge about science, visual arts, culture, history, and mathematics (Kayaalp et al., 2024; Andre et al., 2017). In recent years, museums have experienced significant transformations. They have more diverse connections with society and serve as inclusive and global cultural spaces that reflect new conceptions of culture and the way to access it (González-Herrera et al., 2023). As typical out-of-school educational institutions, museums are globally distributed. They attract large numbers of annual visitors from diverse age groups, including children and young people and enjoy high levels of trust within society and across political contexts (Sutton et al., 2017).

More and more museums are aware of the wide range of their visitors and take measures to make museum settings more accessible for audiences with different backgrounds (González-Herrera et al., 2023; Okvuran and Karadeniz, 2022). As children and young people are becoming core visitors, museums start to serve as important places for informal learning (Tan et al., 2021). Yet such out-of-school resources remain underutilized and child-centered museum programs have not flourished widely (Wong and Piscitelli, 2019). Prior research has emphasized the central role of museum practitioners in shaping learning by selecting exhibition content and organizing educational activities (Hansson and Öhman, 2022; Kristinsdóttir, 2017). However, teachers’ participation has received comparatively less attention than that of parents, professionals, and curatorial staff (Kletchka, 2021; Callanan et al., 2020). Moreover, it is challenging to assess young children’s learning through museum settings and most studies have focused on museum visits of K-12 students rather than younger groups such as preschoolers and primary school students (Kletchka, 2021; Andre et al., 2017). Although many teachers express willingness to engage in related training, few opportunities are provided for them to do so (Noble, 2021). Furthermore, external factors such as policies and support from school management have a strong influence on teachers’ intention to implement class visits to museums (Wu, 2024). Yet little attention has been paid to how internal factors, including teachers’ perceptions, or how internal and external factors together affect teachers’ engagement in museum education for children.

## Museum education: informal and free-choice learning

Museum education is commonly conceptualized as a form of informal or free-choice learning and it emphasizes learners’ autonomy and engagement with objects and narratives (Falk and Dierking, 2016; Hooper-Greenhill, 2007). From a constructivist perspective, learners construct their own meanings and interpret learning opportunities in their own ways (Hooper-Greenhill, 1999). Based on that, learning in and from museums is more related to the meanings that visitors choose to make of the museum experience (Falk et al., 2006). Such characteristics distinguish museum education from the type of compulsory learning that occurs in schools. When museum education is implemented through school-organized visits, potential tensions may arise between formal schooling and free-choice learning. Teachers are typically responsible for student safety, discipline, and curriculum alignment (Souza et al., 2023; Shaby et al., 2019; Karnezou et al., 2013), which can constrain opportunities for open exploration and self-directed learning. Therefore, museum education in school contexts is shaped by school culture, policy requirements and teachers’ professional experiences.

In this study, the term museum education mainly refers to school-led museum visits and related educational activities as understood and enacted by teachers. Teachers’ interpretations of museum education are examined not as theoretical representations of museum learning, but as practice-based understandings that emerge based on personal experience and broader sociocultural contexts. By highlighting teachers’ perspectives, this study seeks to illuminate how museum education is conceptualized in school practice in China, and how these interpretations interact with both internal factors and external conditions.

## Bronfenbrenner’s ecological systems theory

To address these gaps, the theoretical framework of this study is based on Bronfenbrenner’s ecological systems theory. Bronfenbrenner (2000, 1979) pointed out that environments play an important role in children’s learning and development and emphasized the importance of understanding both the objective qualities and meanings of environments for the child in the setting. According to ecological systems theory, children’s development is closely related to how they interact with their surroundings (Crawford et al., 2020). Accordingly, children should be provided with educational opportunities that help them make sense of their environments (Saracho, 2023; Hammar Chiriac et al., 2023).

There are five interconnected systems distinguished according to ecological systems theory: chronosystem (e.g., broader societal changes over time), macrosystem (e.g., cultural values or teachers’ implicit biases), exosystem (e.g., state and educational policy), mesosystem (e.g., interactions between teachers and families), and microsystem (e.g., classrooms). Implementing museum education requires close collaboration between environments (including schools, families, and museums) and the interactions within these environments directly influence children’s learning outcomes (Miller, 2016). Therefore, applying ecological systems theory helps enhance our understanding of museum education by linking external factors (e.g., policies, institutional support) and internal factors (e.g., teachers’ experiences, emotions, and cognitions) within different environments. This also provides insight into children’s museum education from teachers’ perspectives and how different factors affect its implementation in China.

## Methods

### Grounded theory

This study utilized the Straussian version of grounded theory, which is widely adopted in educational research (Stough and Lee, 2021). The researcher analyzed data comprehensively and systematically and establish connections among concepts with high accuracy and complexity through grounded theory (Corbin and Strauss, 2014). Accordingly, this study employed grounded theory with semi-structured interviews to obtain authentic insights into children’s museum education. The collected data were coded in three stages and the researcher extracted core concepts from the interview transcripts by using NVivo 15. Ultimately, a model of factors influencing children’s museum education from teachers’ perspectives was constructed. This not only enhances the explanatory power of the model but also offers a novel perspective on museum education for children.

### Research site and participants

In recent years, China has experienced a museum boom, which has expanded both the number of institutions and the scope of educational programs (Yu, 2025). Museums in China are generally categorized into four types: historical, art, science and technology, and natural museums, with historical museums being among the most significant and popular categories (Zhang and Courty, 2021). Henan Province is recognized as a national leader in cultural preservation and promotion (Wu, 2024), and more than 400 museums are located here (The People’s Government of Henan Province, 2025). Therefore, Henan Province was selected as the research site for this study. Given that many museums in Henan emphasize regional culture, antiquities, and history (Chen and Ryan, 2020), this study focused on children’s museum education in historical museums.

Considering the importance of museum education for young children, who experience less academic pressure and have more opportunities to engage in such activities, two main criteria guided participant recruitment. First, both preschool and primary school teachers were sought in order to cover children across different educational levels. In this study, the term “preschool” refers to educational institutions for children aged three to six. Second, consideration was given to teachers’ level of teaching experience. Purposeful sampling (Suri, 2011) was employed to select participants from preschools and primary schools located in both rural and urban areas of Henan. Based on these criteria, 20 preschool teachers and 25 primary school teachers were recruited, all of whom were individually interviewed after providing informed and voluntary consent (see Table 1).

**Table 1:** Sample distribution (total = 45).

Characteristic	Categories	Frequency	Percentage
Gender	Female	41	91.11%
	Male	4	8.89%
Schooling stage	preschool	20	44.44%
	primary school	25	55.55%
Discipline	Chinese	32	71.11%
	Ethics and the rule of law	5	11.11%
	Music	4	8.89%
	Art	4	8.89%
Museum education (per year)	Never	7	15.56%
	Once or twice	29	64.44%
	More than 3 times	9	20.00%

### Data collection and analysis

To ensure the collection and analysis of data within authentic problem contexts, this study followed the principle of theoretical sampling. Face-to-face semi-structured interviews were conducted in May 2025, with the interview outline designed according to the research purpose (as shown in Table 2). Each interview lasted between 21 and 49 min. Sampling continued until no new categories emerged and theoretical saturation was achieved. All interviews were audio-recorded and transcribed verbatim by the researcher. Ethical approval was obtained from Zhengzhou University of Science and Technology, as well as from the principal of the participating school. To maintain confidentiality, pseudonyms were used for all participants.

**Table 2:** Interview outline.

Sequence	Main interview questions
1	Could you describe your understanding of museum education for children?
2	Can you recall your experiences with museum education during childhood, and explain whether they have influenced you today?
3	Can you talk about your teaching experience in designing or engaging in museum education/
4	How do you feel when participating in museum education?
5	What would you recommend for a new museum visit?

The study adopted the three-level coding strategy proposed by Corbin and Strauss (2014) and employed NVivo 15 in order to systematically read and analyze the interview data. Through open coding, axial coding, and selective coding, the researcher analyzed the data in a bottom-up manner (Liu and Zhang, 2024). A comprehensive model was constructed to illustrate teachers’ perceptions of museum education for children after the theoretical saturation was confirmed.

### Open coding

The initial stage of data analysis was open coding and it included reading the interview transcripts, segmenting the data, assigning descriptive labels, extracting concepts, and developing categories (Corbin and Strauss, 2014). There were 25 initial concepts identified after removing irrelevant materials and consolidating redundant codes. Through comparative analysis, nine broader categories were abstracted. The results of the open coding are summarized in Table 3.

**Table 3:** Open coding table.

Axial coding	Open coding	Reference point content examples
Activity experience	Subjective perception	Not necessary; Meaningful learning; Important for children
	Convenience	Limited room to participate; Time-consuming approval; External coordination difficulty; Inconvenient arrangements; Limited access in rural areas
Personal experience	Visit experience	Simplified visits; Need more engaging exhibitions;
	Professional background	No museum-related knowledge in prior education; Lack of academic preparation;
Personal cognition	Professional abilities	Insufficient skills; Difficulty in designing tasks
	Knowledge structure	Limited interdisciplinary knowledge; Collections unfamiliar
	Self-identity	Classroom educator; Uncertain role; Not museum educator
Perception barriers	Negative perception	Not effective; Too difficult to manage; Less fun than amusement parks; Ritualized procedures; Sense of failure
	Lack of assistance	Explore on my own; Rarely arrange visits; Feel unsupported from staff;
	Children’s security	Too young to go to crowded places; Worry about safety
	Content selection	Too profound for children; Lack of child-friendly exhibition design; Difficulty in understanding
Internal motivation	Participation mentality	Supportive and treat as exposure
	Self-efficacy	I cannot be a good guide; Low confidence to organize museum visits
	Professional identity	I believe I can help children; Role adjustment; Professional responsibility in museum education
School support	Curriculum integration	Lack systematic curriculum design; Well-planned programs
	Leadership attitude	Principals decision matters; Layers of approval from management
	Logistical support	Funding for museum education; Arrangements well-planned
	Training implementation	No in-service training provided; Lack of practical workshops;
Family support	Family concept	Decisive to museum education; Do not recognize value
	Financial support	Reluctant to afford expenses
Museum resources	Appropriate resources	Insufficient resources for children; Lack of interactive tools
	Professional staff	Little help from staff; Lack of trained museum educators; Absence of child-focused docents;
Museum policies	Funding policy	Lack funding support; Sufficient funding needed
	Government policy	Museums unevenly distributed; necessity of clear standards
	Teacher training policy	Policy lacks concrete training measures; Need future training policies

### Axial coding

Axial coding constituted the second stage of analysis. During this stage, the researcher aimed to clarify the relationships between concepts and categories based on the results of open-coding (Stough and Lee, 2021). After examining the nine categories recognized in the first stage, there were four core categories identified: outside environment, institutional support, teachers’ experiences, and cognitive–emotional aspects.

### Selective coding

**Figure 1:**
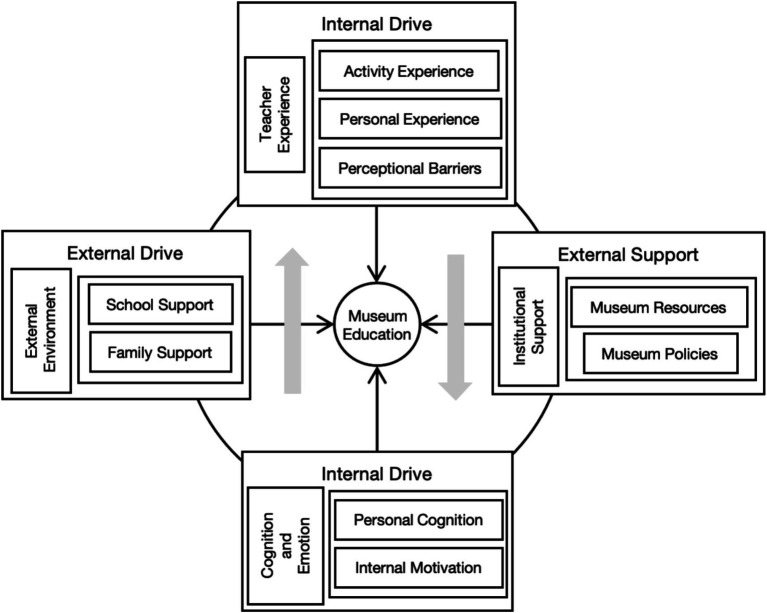
Children’s museum education model from teachers’ perspectives.

As the final stage of analysis, selective coding aims to specify the connections between the core categories and the main categories (see Table 4). This study primarily concentrated on teachers’ perceptions of museum education for children. Comparative analysis indicated that external objective conditions (e.g., external environment and institutional support) and internal subjective factors (e.g., teacher experiences and cognitive–emotional aspects) constitute the primary influences shaping teachers’ practices in museum education. Each core and subcategory was described and illustrated, with labeling to the original data if necessary. To ensure the credibility and trustworthiness of the analysis process, peer debriefing (Creswell and Creswell, 2017) and an inquiry audit by two scholars were conducted to refine coding decisions, verify English translations, and confirm the accuracy of the findings (see Figure 1).

**Table 4:** Axial coding table.

Core category	Axial coding	Meaning
Teacher experience	Activity experience	Teachers’ experiences in organizing and engaging in museum education activities
	Personal experience	Teachers’ individual experiences with museums in their personal growth and learning that influence their implementation of museum education
	Perception barriers	Perceived obstacles that prevent teachers from implementing museum education
Cognition and Emotion	Personal cognition	Self-understanding gradually constructed by teachers during museum education
	Internal motivation	Teachers’ intrinsic driving forces of participating in museum education
External environment	School support	Support from schools for museum education
	Family support	Family attitudes and resources that affect children’s participation in museum education
Institutional Support	Museum resources	Educational resources and staff support provided by museums to assist museum education
	Museum policies	Macro-level policies such as governmental and institutional policies that create structural support for museum education

## Results

### Teacher experience: an internal support for museum education

Teacher experience refers to teachers’ overall experiences related to museum education. It encompasses their professional competence, personal background, and prior exposure to museums, all of which shape how they implement museum education. Positive experiences often strengthen teachers’ willingness to participate in museum education, whereas negative ones can hinder their motivation (Yue et al., 2024). Understanding these intrinsic connections is therefore essential. Teacher experience in this study can be organized into three dimensions: activity experience, personal experience, and perceptional barriers.

### Activity experience

In this study, teachers’ activity experience refers to their subjective perceptions of children’s museum education and it includes two main aspects: subjective perceptions and convenience. According to the interview transcripts, many teachers believe that museum education is “*a meaningful learning opportunity for children*” and “*important to promote children’s development*” (T12, T2). “*Implementing museum education helps children have more access to new information and knowledge. Some knowledge or exhibits which are difficult to present in class can be shown in a more vivid way through museum education*” (T6). Furthermore, some participants think museum visits have positive effects on establishing children’s cultural identity and sense of belonging. One teacher mentioned, “*Children can not only feel the cultural atmosphere but also strengthen their cultural confidence*” (T7). However, according to the interview transcripts, some teachers are hesitant to implement museum education. One teacher explained, “*I almost have no opportunities to decide which museum to visit or how to organize museum education. I just follow what the school management orders and this makes me feel powerless*” (T9). This is in alignment with the findings of previous research that school settings can strongly shape teachers’ practices (Khaleel et al., 2021).

### Personal experience

Teachers’ personal experiences, in this study, refer to teachers’ previous personal experiences related to museum visits or museum education, including their museum visits when they were young, related educational background and professional experiences. Previous research indicated that teachers’ personal experiences (e.g., life experience) had a significant effect on teachers’ teaching philosophy and practices (Gao et al., 2024; Kacetl and Klímová, 2021). For instance, one participant mentioned, “*When I was young, I went to museums several times. These experiences left a deep impression on me and developed my interests to Chinese culture. As a teacher, I would like to create similar opportunities for my students.*” Some teachers also expressed the positive influence that training has on their organization of museum education. For example, T4 mentioned, “*I learned theories about kindergarten field trips such as museum visits and how to organize them through university courses. During this process, I felt the importance of out-of-school educational activities. So, I really want my students to have experiences of those activities. In museum education, teachers need guide and allow children to experience it firsthand*.” Such examples illustrate how personal experience can foster teachers’ commitment to museum education.

### Perceptional barriers

Despite the benefits, teachers have encountered various obstacles of implementing museum education. Museum visits are common means for teachers to implement museum education and educators express great concern about student safety during the visit (Krantz and Downey, 2021). As mentioned by some respondents, “*Every time we go on a museum visit, the number of children makes supervision and management challenging. I rarely have time to focus on what children are actually learning. The procedures of these activities are simplified. Some visits are generally limited to nearby museums for practical and safety reasons*” (T2, T13, T17). Furthermore, professional challenges also surfaced and influenced the implementation of museum education. “*Museum education covers a wide range of knowledge, which creates certain difficulties for me. Sometimes I cannot answer children’s questions*” (T9). Teachers’ anxiety and pressure can be increased if they lack related interdisciplinary knowledge (Yue et al., 2024). According to the interview data, it was revealed that student management was another challenge for museum education. As one teacher explained, “*When we take children to museums, it usually takes a whole day. Children tend to be restless and distracted especially in the end of the activity. At that time, it is difficult for me to effectively guide children and help them actively participate in activities.*”

### Cognitive emotion: internal driver of museum education

#### Personal cognition

Personal cognition in this study refers to the self-awareness that teachers gradually develop through museum education. From interviews transcripts, it can be seen that some teachers positioned themselves as “all-rounders” and took responsibility for imparting knowledge, offering support and ensuring students’ safety. One teacher mentioned, “*Although I notice I need to impart knowledge and ensure children’s safety during museum education, I am an ordinary person and my capabilities are limited*” (T21). Other teachers tend to be “guiders” to “help children develop interests and interact with exhibits and culture” (T9, T23). However, teachers sometimes doubt their own abilities and suffer from pressure due to lack of support. Some teachers mentioned that managing a “large group of students” and imparting “interdisciplinary learning content to children” are challenging (T20, T3). These pressures make teachers rethink the roles they play in museum education. “*There are many potential risks, especially during field trips. My role usually becomes a ‘repeater’ to remind children to stay safe and reinforce discipline*” (T34). Some teachers even view themselves as “safety supervisors” (T16) or “actors” (T19). This highlights the tension between different teachers’ roles that they play when implementing children’s museum education. The findings are in accordance with the results of prior research which demonstrated that teachers’ wellbeing and psychological competence were closely linked to the internal and external support (Nwoko et al., 2023).

#### Internal motivation

Internal motivation refers to teachers’ intrinsic drive to engage in museum education for its own value rather than external rewards (Fishbach and Woolley, 2022). Interviews and analysis results revealed that teachers’ motivation for museum education was closely tied to teachers’ emotional perception, participation, self-efficacy, and professional identity. While some teachers expressed enthusiasm, many also reported exhaustion and anxiety. For example, T2 shared, “*When there are so many children to manage, I worry constantly. If I just need to keep them safe, I feel satisfied.*” Moreover, teachers may feel pressure when the learning activities were organized by others. “*If other teachers design the museum education, it is challenging for me to engage in because I have to balance my roles during learning activities*” (T4). Furthermore, from the interview transcripts, it was found that teachers with more confidence in their abilities were more likely to implement field trips while teachers with lower self-efficacy preferred classroom-based learning. “*I would like to stay in the classroom where I feel competent*” (T5). “*Being a subject-focused teacher is already enough for me*” (T9). This aligns with the findings of prior research which indicated that teachers’ self-efficacy has a significant impact on teachers’ practices and their level of self-efficacy varies across different activity domains (Woodcock et al., 2022). Based on those findings, it is clear that teachers’ cognitions and emotions (e.g., self-perceptions, intrinsic motivations) are powerful internal drivers which affect not only how teachers participate in children’s museum education but also how they deal with challenges during it.

### External environment: contextual drivers of museum education

#### Support from schools

School support is closely related to teachers’ motivation and intention to persist in learning activities (Lam et al., 2010). Insufficient support often hinders teachers’ effective implementation of museum education. As one participant explained, “*While schools handle most aspects of organizing museum visits, I have few opportunities to participate in decision-making. I need to get approval from school management for many times, and such a process makes me feel a sense of failure*” (T27). Moreover, school support shows disparities between rural and urban areas. “*Rural schools and kindergartens usually lack systematic and comprehensive curricula. There was limited funding for out-of-school learning activities and related teacher training. As a rural teacher, such a situation makes me feel that museum education is not necessary for children’s learning*” (T15). In contrast, “*When school arrangements are well-planned, it can help me build distinctive learning programs for children*” (T2). These findings suggest that school support not only affects teachers’ intention to organize museum education but also influences their long-term persistence of it.

#### Family influence

Family involvement is integral to the success of museum education (Morris and Taylor, 1998). “*Parents’ attitudes and support are decisive to museum education*” (T14). Some parents were supportive and regarded museum visits as valuable learning opportunities (T1, T2, T4), others expressed hesitation and their concerns about children’s safety, age, and financial cost. “*Some parents think their children are too young to go to crowded public places. Some are reluctant to afford the expenses of museum visits, so they seldom let their children participate in such out-of-school activities*” (T16). Another barrier was the lack of parental recognition of museum education’s educational value. “*Some parents do not recognize the value or significance of museum education. They feel taking children to museums is less fun than going to amusement parks*” (T12). From those findings, it can be seen that parents’ attitudes directly or indirectly influence children’s participation and teachers’ confidence in implementing museum education. This supports prior findings that family support shapes both student outcomes and teachers’ self-efficacy (Stipek, 2012).

### Institutional support as an external enabler

#### Museum resources

Museum resources have increasingly become essential for both scientific research and education (Li et al., 2022). High-quality, child-oriented museum resources can enhance learning experiences, help children better grasp and apply knowledge, and improve learning efficiency. The majority of participants emphasized that museum resources play a vital role in the development of museum education and expressed the desire to integrate them into “daily teaching” and “curricula” (T4, T27). Nevertheless, some limitations of current available museum resources were also highlighted both in prior research and through interviews. Zakaria (2024) pointed out that museum programs were limited and insufficient and it is challenging for teachers to integrate museum resources (e.g., collections and exhibits) into school curricula. One participant explained: “*There are insufficient resources specifically for children. Most exhibits are too profound and unattractive to children. In most cases, children have difficulty in understanding*” (T2). Facing this situation, one respondent mentioned “*I have difficulty in guiding children to understand the knowledge from those resources. Docent explanations and interactive facilities for children are needed*” (T14). Disparities in access further compound the challenge. “*Museums are unevenly distributed and there are far more museum resources in big cities. Rural schools have limited access to museum resources*” (T3). Based on the participants’ narratives, we found that, due to lack of sufficient and child-appropriate museum resources, museum education was generally limited and some museum visits were even “simplified” (T18), resulting in the ritualized procedures of museum education and reducing their educational value.

#### Museum policies

Policy frameworks and clear standards are important to the development of children’s museum education. Zakaria (2024) demonstrated that supportive policies can not only enhance children’s learning experiences during museum education but also cultivate their cultural identity and sense of belonging. However, many teachers noted that museum education is not compulsory in their schools. It usually receives limited funding and weak regulatory support. “*Although it is meaningful to be involved in museum education, I believe I can help children have a better learning experience if there is sufficient funding and museums*” (T3).

Developing children’s museum education needs to deal with problems of staff shortage and lack of professional training. “*Sometimes I feel restless and exhausted when implementing museum visits. I receive little help from museum staff*” (T7). Another teacher added, “*Kindergartens rarely arrange museum visits. As a teacher, I never received relevant training about museum education. I have to explore how to design and organize a museum visit on my own*” (T19). Those findings reflect broader systemic issues. It echoes the results of prior research about the need for policymakers to balance equity and efficiency in expanding access to museums (Avgousti and Papaioannou, 2023; Chang et al., 2021). Through the interviews, it is clear that many participants underscore the importance of a more comprehensive policy framework for museum education. Such a framework could provide stronger institutional support and enable teachers to implement museum education more effectively and sustainably.

## Discussion

The findings indicate the role that teachers’ perceptions of museum education play in shaping how museum learning is understood and enacted in school contexts. The divergence between these practice-based understandings and common conceptions of museum education in theoretical literature provides an important lens for interpreting the results. As shown in Figure 1, the results demonstrate that there are four core categories identified that influence children’s museum education from teachers’ perspectives, including teachers’ experiences, cognitions and emotions, external environment and institutional support. This is in line with Bronfenbrenner’s social-ecological framework that individuals’ development is closely related to institutional and social factors. In China, current national curriculum reform and central government incentive policies highlight the important role of museums in children’s education (Yue et al., 2024; Chang et al., 2021). Whereas prior research mainly focused on the effects of educational reform on museum education through longitudinal designs (Crispin and Beck, 2025; Monteagudo-Fernández et al., 2021; Datnow et al., 2023; Zheng et al., 2021), this study employed grounded theory to address the research question: How does museum education develop within a multidimensional social context? The theoretical model of this study uncovers the mechanisms underlying children’s museum education and enriches the literature with a dynamic explanatory framework.

### Teachers’ conceptions of museum education

The findings reveal a clear difference between teachers’ practical conceptions of museum education and those reflected in theoretical literature. Most teachers believe that the primary type of museum education in school contexts is school-organized visits aimed at enriching knowledge and supporting curricular goals. Such understandings emphasize structural participation, collective discipline, and predefined educational outcomes. In contrast, museums are commonly conceptualized as free-choice learning environments, where learning is driven by visitors’ interests, motivations, and meaning-making processes (Falk et al., 2006; Hooper-Greenhill, 1992). From this perspective, museum education is not defined by what is taught, but by what learners choose and how they interpret their experiences.

This divergence suggests that when teachers recognize the educational value of museums for children, their practice-oriented conceptions tend to privilege schooling over learner autonomy, which reflects the constraints that school-based museum visits often face. The findings therefore highlight a key tension between the principles of free-choice learning and the realities of formal schooling in school-led museum visits.

### Enhancing teacher experiences and cognitive-emotional engagement

The results of this study revealed that internal factors such as teachers’ experiences, cognitions and emotions jointly influenced their implementation of museum education for children. On the one hand, positive and meaningful school or personal museum visits or effective opportunities to guide children can enhance teachers’ confidence in organizing museum education. On the other hand, barriers (e.g., large class sizes, low participation in decision-making and insufficient interdisciplinary knowledge) can reduce teachers’ motivation and often generate teachers’ anxiety or self-doubt. These findings echo the results of prior studies which indicate that teacher beliefs, prior experiences, and affective dispositions play a decisive role in shaping classroom practices (Gao et al., 2024; Karnezou et al., 2021). They also align with previous studies that self-efficacy influences teachers engagement in innovative practices (Woodcock et al., 2022). Furthermore, the findings of this study highlight the interaction between teachers’ experiences and their emotional states. Negative experiences (e.g., difficulties and barriers during museum visits) can undermine teachers’ self-efficacy and reduce their willingness to participate. Therefore, teachers’ training should not only enhance their interdisciplinary knowledge required in museum education but also focus on teachers’ emotional resilience and self-efficacy. Measures (e.g., professional learning communities, mentorship, reflective practices) can help teachers integrate positive experiences into their engagement with museum education.

### Strengthening the external environment through schools and families

From this study, it is clear that external environments, particularly support from schools and families, are important to the implementation of museum education. Teachers’ motivation can be encouraged with well-planned institutional support, but excessive procedures or lack of resources from schools limits their participation in museum education. Similarly, families’ involvement varies in museum education. The results demonstrate that some parents believe that museum visits were valuable for children’s learning, while others express concerns and are reluctant to let their children participate in museum visits. These findings are in accordance with results of prior studies that institutional support and parents’ involvement influence teachers’ practices (Stipek, 2012; Lam et al., 2010). The results also extend previous research by showing how school and family factors affect teachers’ willingness to organize museum education. According to the results, we found that improving museum education required a high quality external environment and strong school-family-museum partnerships. For schools, it involves reducing approval-related barriers, providing sufficient funding and ensuring teachers’ participation in decision-making. For families, it means raising parents’ awareness of the value of museum education. Measures such as increasing communication and outreach programs can be taken to develop parents as collaborative partners in children’s museum education.

### Optimizing museum resources to support education

Museum resources are increasingly necessary for children to have access to cultural heritage and provide them with unique learning opportunities (González-Herrera et al., 2023; Karnezou et al., 2013). Many museums offer tactile tours and hands-on workshops to enhance audiences’ visiting experiences (Cavazos Quero et al., 2021). The results of this study demonstrated that teachers valued museum resources but they often struggled with difficulties and barriers (e.g., limited professional assistance and guidance, uneven museum distribution and lack of child-centered design).

Through interviews, teachers expressed difficulties they encountered in integrating museum resources into school curriculum and highlighted the need for docent explanations and interactive facilities designed for children. Furthermore, the disparities in museum distributions between rural and urban areas further restricted access and this limited rural teachers’ implementation of museum education for children. Previous research also pointed out that there were structural inequalities in access to museum resources between different areas (Zakaria, 2024; Chang et al., 2021) and the challenges of integrating museum education into school curriculum (Avgousti and Papaioannou, 2023). Emerging evidence suggests that online and digital museum resources can provide virtual access to cultural heritage and mitigate such disparities (Li et al., 2022). Accordingly, policies and museums should focus on optimizing museum resources, developing exhibits or activities tailored for children, and offering effective training for teachers and museum practitioners to facilitate the integration of resources into classroom practices. By addressing both material and human resource gaps, museums can become more inclusive and accessible educational partners.

### Improve policy framework

In recent years, policy discourse related to museum education has attracted increasing attention. In 2019, the International Council of Museums (ICOM) accepted that museum exhibitions and related educational programs can promote and empower teaching and learning and are influenced by policies (Hansson and Öhman, 2022). A supportive policy framework can establish strategic foundation for open museum education so that the external and internal museum practices and educational programming can be designed in an inclusive and co-created way (Atenas et al., 2020). This study found that Chinese museum education is a continuous and dynamic process that is influenced by multidimensional social contexts. Governments and educational institutions should develop policies and strategies to bridge school and museum educational activities (Feliu-Torruella et al., 2021). Accordingly, this study contributes by highlighting the need for policy frameworks that move beyond temporary incentives toward long-term institutionalization. Such frameworks should ensure equitable allocation of resources across urban and rural contexts. Moreover, structured professional development for teachers and museum personnel should be provided. It is also urgent to integrate museum education into broader curriculum reform. Through offering a clear and coherent policy context for children’s museum education, governments can enhance its sustainability and reinforce its role in children’s learning.

## Conclusion

This study concentrates on children’s museum education in China from teachers’ perspectives. The results found the difference between teachers’ perceptions and common definition of museum education and revealed that teachers’ implementation was influenced by both external and internal factors. Institutional support and the broader social environment provide the structural conditions for museum education. Internally, teachers’ experiences and cognitions and emotions have effects on their willingness and capacity to implement museum education. Based on grounded theory approach, this research constructed a theoretical model that illustrates how these four domains interact and influence teachers’ engagement in museum education.

The study, to some extent, extends Bronfenbrenner’s ecological systems theory to the context of museum education. On the one hand, it highlights how external and internal factors influence teachers’ implementation of museum education in China. On the other hand, it deepens the understanding of practices and challenges of children’s museum education through teachers’ voices. Furthermore, this study underscores the importance of policy frameworks and museum resources to museum education. Measures such as providing digital and social platforms and enhancing teachers’ training can be taken to improve the accessibility and quality of children’s museum education.

However, this study also has some limitations. First, as the study adopted a qualitative grounded theory approach, the sample size was determined by theoretical saturation rather than statistical representativeness. The limited number of male participants mirrors the demographic composition of early childhood and primary education in China. The small sample size and gender imbalance of participants may affect the transferability of the findings. Future research can use a larger or mixed-method sample to further examine the patterns identified in this study across broader populations. In addition, although this research adopted the qualitative approach and provides insights into museum education for children from teachers’ perspectives, interpretive bias may occur due to the reliance on subjective accounts. Therefore, further studies may integrate quantitative methods to strengthen the robustness of the conclusions.

## Data Availability

The original contributions presented in the study are included in the article/supplementary material, further inquiries can be directed to the corresponding author.
